# An International Survey on Olive Oils Quality and Traceability: Opinions from the Involved Actors

**DOI:** 10.3390/foods11071045

**Published:** 2022-04-05

**Authors:** Ramón Aparicio-Ruiz, Noelia Tena, Diego L. García-González

**Affiliations:** 1 Departamento de Química Analítica, Facultad de Farmacia, Universidad de Sevilla, Prof. García González, 2, 41012 Seville, Spain; aparicioruiz@us.es (R.A.-R.); ntena@us.es (N.T.); 2 Instituto de la Grasa (CSIC), Campus Universidad Pablo de Olavide, Edificio 46, Ctra. de Utrera, Km. 1, 41013 Seville, Spain

**Keywords:** olive oil, survey, traceability, quality, protected designation of origin, best before, sensory assessment

## Abstract

A survey was launched to understand the current problems and sensitivities of the olive oil market through a series of questions clustered around topics related to quality, traceability, regulation, standard methods and other issues. The questions were selected after a series of interviews with different actors to identify those aspects where some disagreement or different points of view may exist. These questions were grouped in topics such as geographical traceability, consumer perception and quality management. The survey was addressed to eight different olive oil actors independently: producers, retailers, importers, exporters, analysts, workers at regulatory bodies, and consumers. Approximately half of the respondents (67.0% for consumers and 56.0% for the rest of olive oil actors) claimed to understand the importance of the protected designation of origin. In fact, the traceability objectives that were selected as the most relevant were those related with geographical traceability (19.3%) followed by the detection of adulteration (15.6%). Most of the respondents (80%) would agree to share data for a common database; however, some concerns exist about the use of these data and the issue of paying to have access to this database. The respondents mostly expressed an affirmative answer concerning the efficiency of panel test (74%) and a negative answer (90%) concerning the proposal of removing from regulation, although 42% agree with their revision for improvement. The opinions on “best before” date and their relationship with quality and the willingness to apply non-targeted methods were also surveyed.

## 1. Introduction

Olive oil production has been traditionally associated to the producing countries of the Mediterranean Basin, mainly Europe, North Africa and Turkey. However, a few decades ago, the production in areas of America and Australia, which was residual up to that time, started to increase making use of new agriculture practices (‘super intensive’ and ‘super high intensity’ orchards) and highly adaptable cultivars (e.g., Arbequina, Arbosana, Koroneiki, Leccino, etc.). Thus, the direct association of olive oil production to Mediterranean area is not completely accurate today. In fact, 10 million hectares of olive groves are distributed in the world [1], including countries as far flung as New Zealand, USA, South Africa and Chile. These countries have areas within them with pedoclimatic conditions similar to those in the Mediterranean basin.

On the other hand, in the last two decades, the olive oil market has been subjected to a series of changes that are still in constant evolution today: (a) The production of olive oil has shown a rising trend in most of producing countries—both Mediterranean and non-Mediterranean—[2]; (b) the number of consumers (habitual and newcomers) has also increased because of publicity programs and a major attraction of consumers to healthy products; (c) although International Olive Council (IOC) is the reference organisms for trade standards and standard methods [3] and the European Union has its specific regulation mostly aligned with the IOC norms [4], new regulatory bodies have come into play or have reinforced their role in controlling olive oil quality and authenticity [5,6]; (d) additionally, some national regulations of new-producer countries, which have traditionally ignored the olive oil market, have now included specific provisions in their regulations on olive oil [6]; (e) the perfection of the testing methods for detecting classical adulterations has allowed paying more attention to sensory quality, which has been identified as an added value for increasing competitiveness [7]; (f) the different criteria for guaranteeing quality and the influence of new climates on olive oil composition [8] have led to an update of some national regulations and a consequent heterogeneity in international regulation [6]; (g) the mass media has contributed to a major knowledge of consumers in cooking uses of olive oil and they also reported on quality characteristics, thereby influencing consumers’ opinions and dynamizing discussions in regulatory bodies and scientific and technical fora [9]; (h) finally, the spread of production in different places has diverted the business models with a consequent lack of homogeneity in economic interests and different degrees of consumer protection [10]. The importance of these factors needs to be considered in the context of the complex and variable composition of olive oil, which is affected by pedoclimatic conditions, variety and harvest year [11,12]. This composition of olive oil is made up of triglycerides and fatty acids [13,14], fatty alcohols [15], sterols [16], waxes [17] and other minor compounds such as tocopherols, phenolic compounds, carotenes, chlorophylls, n-alkanes, n-alkenes and volatiles with different implications in quality and authenticity [5,18,19]. These chemical compounds may change according to cultivar, geographical area of production, microclimate, soils, fertilizers, irrigation, extraction system and storage conditions [5,7]. Thus, different technical and marketing aspects in discussions of olive oil also have some chemical implications that affect olive oil analytics, traceability and quality control. Regarding quality, it is important to note that virgin olive oil, unlike other vegetable oils, is subjected to tasting control through a standard method of organoleptic assessment that is internationally applied [3,20]. Volatile compounds are responsible for the aroma and therefore for the sensory attributes perceived by consumers [9,19], and they are an example of minor compounds with major sensory impact.

Given recent trends in olive oil marketing, it is also important to consider the factor of consumer awareness about the nutritional properties of this foodstuff overall, and to compare virgin olive oil categories with other refined seed oils. It is well known that virgin olive oil is one of the main ingredients that give the Mediterranean diet its health benefits. The nutraceutical properties of virgin olive oil have been demonstrated in multiple studies [21,22,23,24,25]. Monounsaturated fatty acids and several phenolic compounds, such as oleocanthal, oleuropein, hydroxytyrosol and tyrosol are among the main bioactive compounds in virgin olive oil. It has been suggested that the nutraceutical components of virgin olive oil have the capacity to modulate the processes associated with ageing. Thus, high concentrations of polyphenols have anti-inflammatory and antioxidant properties and protect blood lipids from oxidative stress. Oleuropein is a radical scavenger that blocks oxidation of low-density lipoproteins [26] and the fatty acid composition of virgin olive oil is associated with a reduced risk of coronary heart disease. All this knowledge has reached consumers and also affected priorities in quality control, traceability and regulatory actions.

The immediate consequence of all the changes described above is a series of controversial issues, mostly focused on quality and traceability aspects, and a high number of opinions coming from the different actors, some of these opinions even being opposed to one another. Although the discussions on olive oil quality and authenticity are held in stable regulatory and scientific fora, the reality is that it is difficult to record all the opinions from all the actors, basically due to two problems. One of them is the difficulty to conglomerate the opinion of the different sectors. These sectors cover farmers, producers, retailers, importers, exporters, analysts, regulatory bodies, and consumers. Another problem is the difficulty of recording sincere opinions from the private sector, when the image of their product can be compromised. For that reason, within the European Project Food Integrity, it was decided that surveying the opinions from the different olive oil actors would begin. The objective of the survey was to understand the current problems and sensitivities of the olive oil market through a series of questions clustered around aspects related to quality and traceability and their relationship with regulation, standard methods and other issues. For that objective, the answers were anonymous and only the required information was recorded to guarantee the sincerity of the opinions expressed in the survey. The answers have been examined according to the current market situations and topics that are being discussed today. The survey was focused on olive oil in general, although some questions, particularly those related to quality, were related to virgin olive oil. The information extracted from the survey could serve to identify points of consensus or disagreement and could support the importance of open research lines or redefine new quality and traceability strategies.

## 2. Materials and Methods

### 2.1. Survey Launch

The survey was first launched in 2015 on the website of the project (www.foodintegrity.eu, accessed on 31 March 2022) and it was maintained after the project ended, until 2019. The objective of the project was to know the opinion of the eight different olive oil actors independently: farmers, producers, retailers, importers, exporters, analysts, workers at regulatory bodies and consumers. In order to avoid a dispersion of the information among actors, this report shows the results (answers) clustered into five large activity groups, each one having adapted questions: (1) farmers and producers; (2) retailers, importers/exporters; (3) analysts; (4) workers at regulation bodies; and (5) consumers. The survey is presented in Supplementary Materials with all the questions. This article presents the results of the questions related to quality and traceability. Thus, for example, those questions referring to agricultural practices asked to farmers were omitted.

Researchers have analyzed the pros and cons of open-ended and closed-ended questions. We opted for closed-ended questions because of the ease of coding for statistical analysis and interpretation. In order to avoid consumers not understanding the question or not finding a query that accurately reflects their true opinions or attitudes, in each question an additional field titled “would you like to contribute with a free text?” was added. The text that participants eventually wrote in this field helped us to interpret some of the results better.

The following actions were taken to prepare the final version of the survey that was distributed among olive oil actors:

1. The first step was the selection of the questions to be asked to the potential respondents. Thus, a large set of questions were written and discussed among the authors and the olive oil actors from Spain, Italy, USA, Israel and Turkey. Only those questions with enough consensus about their interest were selected. Thus, we moved to different companies and cooperative societies of different geographical origins and sizes where we presented this pool of questions to managers and professionals, and asked them for their opinions. These quite fruitful discussions allowed for selecting the most relevant questions. The objective was to remove those questions that were obvious or that might cause respondents to become discouraged and abandon the survey without completing it.
2. The second action was to translate the survey into the main languages used by the olive oil actors. Thus, the survey was translated into five languages—English, French, Hebrew, Italian and Spanish.
3. The third action was to decide the procedure by which the survey should be distributed. The decision was to disseminate the survey on the project website for all the actors, and additionally for the specific case of farmers as printed-material.
4. The fourth action was to promote the participation in the survey at diverse national and international events, including meetings with farmers/producers (e.g., in Chile, Italy, Israel and Spain), scientific congresses or presentations in different international organizations, in addition to distribute the survey through the project newsletters, web and e-mail communications and social media.

The survey was distributed between the olive oil actors and the percentage of respondents (826) distributed into the different groups of olive oil actors; the strategy followed considered the volume and diversity of opinions. The percentage of respondents for each group was adapted to the size and complexity of opinions in each group. Thus, consumers formed the most numerous group (68%), and due to their high diversity in opinions, it was necessary to increase their number in different countries. The percentage of analysts was the second most numerous (19%), while the group of farmers and producers was lower (11%) and special care was taken to ensure a geographical balance between them. The percentage of workers at regulatory bodies was the lowest (3%).

In terms of the countries that participated in the survey, it was filled in by actors from 30 countries (importers and exporters) from both hemispheres. In the distribution of the survey, the relative importance in production was considered. Thus, a greater contribution from major producers was obtained and special attention was paid to collect enough number of answers from Spain, Italy, Turkey and Tunisia. Likewise, data collection from such major importers as USA, UK, Germany, and France was also considered to be relevant due to its importance in the olive oil world market. The distribution of respondents by country was the following: Italy (390), Spain (179), UK (55), France (31), Germany (19), Portugal (16), Turkey (15), USA (13), Tunisia (9), The Netherlands (9), Israel (8), Argentina (8), Chile (7), Greece (6), China (6), Croatia (5), Cyprus (4), Canada (4), Morocco (5), Switzerland (4), Belgium (3), Jordan (3), Czech (2), Albania (2), Australia (4), Iran (2), Argelia (2), Poland (2), Romania (2), Brazil (2), New Zealand (3), Austria (1), Ecuador (1), while 4 did not declare their countries.

### 2.2. Survey Distribution and Collection of Answers

The format of the survey was a self-administered questionnaire in a computer-based format via KwikSurveys (Bristol, UK). In addition to this online survey, the same questionnaire was also presented on paper, and it was distributed among those olive oil actors that could be reluctant to use the internet. The objective is to avoid any bias because of the survey format, although most of the respondents answered via internet (98%). To obtain a survey with an appropriate length survey, the maximum number of questions was set at 22. A higher number could prevent respondents from answering all the questions. In order to keep the number of questions under this number, the survey included a set of selected questions for all the olive oil actors and another set for the five activity groups already named above. Thus, when a respondent selected the type of olive actor, the survey was adapted with the questions more related with their field, up to a maximum of 22 questions. The data were stored and managed with IBM SPSS Statistics software (Version 27, Armonk, NY, USA) and also with Microsoft Excel 2019.

## 3. Results and Discussion

The total answers were collected and processed as a whole, and they were split into different groups only when a significant difference was observed between sectors or geographical locations. The most remarkable conclusions are reported below clustered into four great groups devoted to knowing the opinion concerning: geographical traceability, consumers’ opinions, sensory quality aspects, and producing new challenges.

### 3.1. Opinions of Olive Oil Actors on Perception and Utility of Geographical Traceability

Some of the questions were selected to extract the opinions about the perception and utility of the geographical traceability, including some provisions of quality schemes associated to geographical identification. Nowadays, it is unquestionable that the provenance of virgin olive oil plays a role in marketing and consumer perception of the product. In fact, extra virgin olive oils from protected designations of origin (PDO) or regional producer zones, in general, have become very popular, with a growing demand for certified quality labels [27,28,29]. Besides, the consumers’ interest towards regional products opened a new marketing strategy for producers and retailers though interests varying according to countries [30,31,32]. Thus, this part of the survey was focused on investigating the perception that olive oil actors have about protected designations of origin (PDOs) and the success of their application over time. The purpose was partially to check the performance of this quality system according to the respondents’ perception and the accumulated experience. The survey asked European consumers, analysts, regulatory bodies, retailers, exporters/importers and producers (539 respondents) their opinion about the PDO concept by means of the following question “A Protected Denomination of Origin (PDO) system differentiates a product from other similar products and therefore it means a protection for certain foodstuffs with a recognized know-how. Do you agree with this statement?”. The analysis of the answers was divided into two groups, the first constituted by consumers and the second by the rest of the actors. The answers of the first group were collected from European olive oil consumers as PDOs concern the European Union only. The results show that consumers—whatever their European nationality—agree with the importance of PDOs for the food sector. In fact, 67% of consumers agree very much with the previous statement, 20% agree a little, 7% do not agree at all and think that PDOs are actually a kind of trademark, and 6% did not answer. This result matches with the consumer perception of virgin olive oil quality that is based not only on the sensory attributes perceived by consumers such as odor, taste, color, but also on other characteristics of the products, such as the origin [33], because consumers tend to prioritize product quality based on their culture [34] up to the point that the certification of origin is one of the main differentiation elements [35,36]. More exactly, virgin olive oils labelled as belonging to PDOs have a greater chance of being purchased [37] as this information is much more important driver for virgin olive oil choice than brand, organic certification and packaging in Italy [38]. However, preferences differ not only among the country of origin [39] but also within the same country [40] since geographical origin has always been considered an essential extrinsic attribute to indicate quality [41]. When price and quality variables are added in reported studies about the attitudes of consumers [32,42], the preeminent importance of PDO is modulated with the price, while the inclusion of PDOs as one of the quality attributes has been widely evidenced in studies about e-commerce [43]. Furthermore, the economic crisis [42] and the incorporation of young people to the market [44] are new factors that have increased the importance of virgin olive oil price in the consumers’ decisions. Thus, the main difference seems to be between normal consumers vs. well-informed consumers, as there is empirical evidence [45] about the role of price/quality factor as a strong predictor of consumer behavior, particularly when the consumer has limited information; it is less important when an intrinsic aspect of the oil (i.e., PDO) is available to consumers. Well-informed consumers are not always associated with producer countries, as a previous survey detected that approximately one-third of the respondents had the misconception that the refining process improves the intrinsic quality and makes olive oil purer [46].

As already stated above, the importance of PDO for other olive oil actors was also analyzed with the answers from producers (large and small), analysts, exporters/importers and regulatory bodies. They showed viewpoints a little more skeptical—50% (very much), 23% (a little), 14% (not at all, it is a trademark) and 13% (contributed with other personal opinions)—that might be consequence of a better information of this set of olive oil actors. Furthermore, within the producers, there are different marketing strategies applied in which the geographical identification may have different importance. Thus, the PDO certification is not always applied by producers, and therefore different opinions were expected. Anyway, a high proportion of olive oil actors think that PDOs are beneficial for either producers or consumers, and PDOs have also boosted the quality control of their extra virgin olive oils.

**Figure 1:**
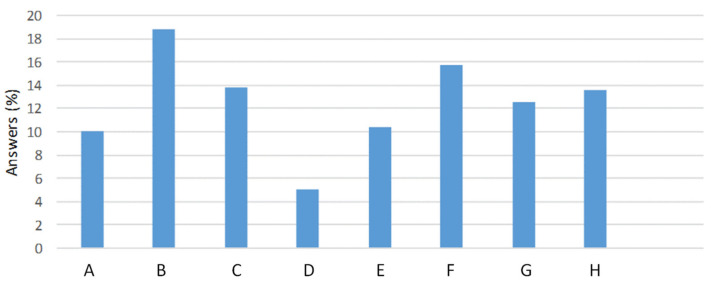
Percentages of the answers (1576) given by farmers/producers, consumers, analysts and regulatory bodies to the eight following questions: (**A**) detection of pesticides and other contaminants; (**B**) geographical identification (identification of production areas); (**C**) detection of critical points affecting to quality; (**D**) reproducibility in the production between crops and extraction systems; (**E**) detection of critical points affecting safety; (**F**) detection of adulteration of olive oil; (**G**) prediction of protection designation of origin (PDO); and (**H**) additional guarantee of the product to gain consumer confidence (competitive product differentiation).

The next question was focused on the expectation that the implementation of a traceability system for virgin olive oil—able to identify from whom and to whom a product has been supplied—which is in force in the European Union [47,48], would have to a better knowledge of the main problems for olive oil actors. In this regard, traceability systems pursue different objectives and it was necessary to know the relative importance of each objective according to the opinion of respondents. This question was asked to all olive actors, with the exception of consumers, as this is a technical topic. Figure 1 summarizes, in percentages, the results of the question: “In your opinion, what are the three main objectives of the implementation of an efficient traceability system for olive oil and virgin olive oil?”. The objectives proposed in this question were previously defined by means of a series of interviews with managers of olive oil producing companies. When the proposed objectives were shown together with this question to the respondents from all the sectors, the answers pointed out that there is not a clear agreement about the most remarkable objectives since the answers were diverse. Thus, the eight objectives proposed to actors were thought to be almost equally important, though with a slightly higher percentage for geographical traceability (19.3%) followed by the detection of adulteration (15.6%) (B and F in Figure 1, respectively). However, the sum of the positive opinions for the importance of PDO and geographical origin (B and G in Figure 1) reached 31.8% followed by aspects related with food safety (A and E in Figure 1) that got 20.6% of the answers. Thus, the importance of quality and authenticity issues were balanced in their importance according to the perception of the respondent. On the contrary, the objective of improving reproducibility in the production between crops and extraction systems was characterized with a particularly low percentage of answers selecting this objective (5%), pointing out that this reproducibility in production is causing relatively fewer problems compared with the other objectives, or they are effectively handled by the producers.

In order to obtain further information about the importance of geographical traceability from the respondent perspective, the next question was “What is the main function a traceability system should perform?” concerning three current important issues: (i) fast detection of adulterated oils to prevent a hypothetical safety crisis in a particular location; (ii) protect traditional production (e.g., PDO); and (iii) distinguish different production areas (e.g., European and non-European oils). The question was asked to producers, analysts and regulatory bodies (136 answers) as they were directly concerned about these topics. The results showed the highest interest for the detection of adulteration (44%), which is not new [7,49], followed by the identification of the producing area (32.5%) due to the combined action of the interest of new producer countries (e.g., USA, Australia, Chile, South Africa) to distinguish their national virgin olive oils from those produced inside the EU and the interest of European large producers (mainly cooperative societies) of identifying extra virgin olive oils imported from outside EU (i.e., Turkey, Morocco and Tunisia) that are next mixed with European oils and sold as “Bottled inside the EU”. The issue focused on the protection of traditional production (e.g., PDO) garnered the interest of 23.5% of the participants in the survey despite PDOs concern EU regulation and not all the respondents were from the EU.

After asking olive oil actors their opinions regarding the effect of an efficient traceability system on solving the main problems of the sector, and given that there is an European regulation and other norms that force traceability systems on food companies, the survey focused on two new aspects: (i) the implementation methodology of a traceability system; and (ii) the level of sharing recorded data—obtained from institutions and companies—in a non-profit network for a better synergy in traceability. The purpose was to check the perception concerning new strategies in traceability control, including database building and managing.

With respect to the implementation methodology, the following question was presented to producers, analysts, exporters, importers, and regulatory bodies: “Given that there is a European regulation and other norms that force traceability systems on food companies, do you think that the traceability system should be centralized in a national/international body?”. Approximately 80% of European actors agree with a centralized system of traceability, although 38% of them considered that this system should be applied in addition to others implemented by companies. In that sense, a significant number of actors (19%) think that each company/producer should implement domestic measures adapted to its capabilities to ensure compliance with traceability requirements.

The same European actors (136) were also asked, with respect to traceability, the question “Would you be willing to provide your recorded data to a network of producers for a better synergy in traceability?”. Unfortunately, only 43% of the respondents would be willing to provide their recorded data since they thought that the exchange of data would be positive for consistency and transparency of the traceability system. The highest percentage (46%) of actors, however, voices that it would depend on the conditions of sharing, which reflects that the intellectual property of the data plays a major role in building common databases or open common spaces for data sharing. In fact, 11% of the respondents answered that sharing information is against their interests. The objective of data sharing is in the line of a better strategy of data management according to the recent European data strategy [50]. Thus, this result in the answers led to a reflection about promoting the benefits of data sharing among producers and other data suppliers if this strategy is implemented.

The survey also asked consumers “are you willing to pay more for a virgin olive oil with additional assurance regarding traceability?”. Although 74% of the 410 answers were affirmative to give economical support to this additional guarantee, 26% disagreed because they thought virgin olive oil was quite expensive already. This aspect would require a specific study since the price sensitivity of consumers depends on many social and economic factors, which also have geographical differences and evolve over time [10,51,52].

The last query about traceability was for retailers, importers, exporters and staff working at regulatory bodies. It was formulated as “With respect to traceability and your opinion, what are the problems of implementing a traceability system in olive oil production? (please, indicate three problems)”, which was answered by 124 actors. The five possible answers—lack of understanding of the use of implementing traceability, lack of analytical tools, lack of guidance on how to implement traceability, costs of implementing the traceability outweigh the benefits, and complexity of the product (number of categories, many actors in distribution)—were selected with similar percentages (range 17–23%), although the complexity of the product (23%), the cost of implementing traceability (21%) and lack of analytical tools (21%) were the most voted by the actors. These percentages pointed out that attention should be paid to these problems with approximately equal priority. Furthermore, these answers were selected after having interviews with producers, which explains the fact that they are problems shared by most of olive oil actors.

### 3.2. How Much Do Consumers Know about Virgin Olive Oil?

The official edible categories of olive oil and olive-pomace oil (extra virgin, virgin, ordinary, ordinary olive oils, olive oil composed of refined olive oil and virgin olive oils, olive pomace oil) [3] for sale in supermarkets and their actual meaning and definition are confusing for most consumers, including consumers from traditionally producing countries [9]. Furthermore, olive oils purchased outside the European Union can be labelled with other grades on their labels like, for instance, pure, light, cold pressing, etc. The high degree of confusion may prevent the access of consumers to this product for use as daily cooking oil. In the survey presented to consumers, basic definitions of the categories olive oil, virgin olive oil and extra virgin olive oil according to the International Olive Council standard [3] were provided to clarify the meaning of the questions (Supplementary Materials). The survey asked the consumers (428) the following question: “When you buy olive oil, do you understand the label with respect to the different quality categories?”. The results showed that most of consumers say that they understand the label (67%). However, a high number of respondents (26%) has some doubts about the meaning of the label in terms of olive oil categories, although the ability of consumers to accurately differentiate the different categories was not tested. Thus, these results should be regarded as the perception of their own knowledge and understanding of the olive oil categories found in the market. When the answers were split into two groups—consumers from producer countries and from main importer countries—it was observed that the percentage of consumers that claimed that they do understand the label was slightly lower (61%) in the second group compared with the first group (67%), which denotes a slightly worse knowledge about the olive oil categories in non-producer countries. In fact, 11% of respondents of the second group recognized that they do not understand the labels versus only 4% of the first group, while the rest (28% and 29%) claimed that they do not totally understand the label and they have some doubts. Lower figures resulted from a survey among 2,234 US consumers [53] in which 55% of the consumers believed that they understood the meaning of olive oil categories, though only 25% responded correctly to statements about the categories. These results can evolve over time depending on promotion campaigns and other factors (education level of consumers over time). Thus, these questions for consumers could be selected to be tracked over the years to redefine actions on consumers and labelling information.

The edible official categories of virgin olive oil (extra virgin, virgin and ordinary) defined by the international trade standard [3] are defined by physical-chemical, chemical and organoleptic characteristics. Although the existence of these categories induces confusion to consumers, some initiatives have suggested a new quality grade beyond the extra virgin category around the world from time to time. The publicized aim is always to provide some confidence to consumers who are regularly informed about stories of olive oil fraud and deceptive messages from industry stakeholders. The survey included the following question to consumers about this matter: “Do you think that a new quality category higher than extra virgin olive oil is necessary or it might bring more confusion?”, which was answered by 414 consumers. The position of the respondents, however, was not clear, as 54% of the consumers think that a new grade would be confusing and it is not needed, while 46% of the respondents think that a Premium extra virgin olive oil grade would be welcomed to prize the oils with the best quality. This disagreement among consumers about their position to support a new grade of extra virgin olive oil had its immediate consequence in the percentage of respondents (18%) who contributed with free texts for explaining their viewpoints. There is a significant number of free texts (37%) that can be summarized in the opinion of a consumer that thinks that “large retailers find no incentive to sell high quality oils, but for those oils widely demanded by consumers”. Other free texts focus on a stricter control of extra virgin olive oils because “extra” does already mean “premium” and “a premium extra-virgin” does not make sense, mostly because the next steps might be “a super-premium extra-virgin” and so on. Some free texts (14% of the respondents) put the emphasis on the knowledge that some consumers have about the categories of virgin olive oil. A consumer summarizes that opinion with the following text: “I believe that the biggest problem is not so much a higher quality category as the consumer awareness which often already confuses the various categories by not paying attention to the presence of virgin and extra virgin adjectives. In addition, many consumers are often used to consume commercial oils of poor quality”. The percentages of the answers and the comments provided denoted a lack of understanding of the actual advantage of establishing a new category of higher quality.

For those consumers that agreed with a new category, the questionnaire also asked whether the hypothetical new category should be included in the labeling regulation or be optional. The objective was to know the opinion about the regulatory implications of this new hypothetical quality grade given that an affirmative answer to the previous question could have different answers regarding the way to implement an additional quality scheme for premium oils. An ample majority (79%) answered that the new category should be included in a hypothetical new regulation while only 9% answered that the new category should be optional and 11% had no a well-founded opinion. One of the consumers explained that they had affirmatively answered to include the hypothetical category in the regulation because “If it isn’t regulated, every company will try to use it without really complying all the constraints” while another one wrote “Regulation would be needed to avoid easy confusion and misunderstandings and as a guarantee for the consumer”. This question was asked without providing further consideration about which quality criteria this new category should have, which is a complex debate.

Another aspect analyzed by the survey was related with labelling, freshness and organoleptic quality. Thus, according to national and international regulations, the labels of olive oil packaging have to include the “best before” term that is related with the concept of “freshness” [3,54,55,56] by a large number of consumers, because it is a word that receives the highest agreement for describing tasty oil [35]. It is well-known among producers that sometimes aged oils from healthy olives can be of better quality than fresh oils obtained from damaged or infected olives. However, consumers usually have a more intuitive or simple perception of freshness [54]. Thus, a question was presented to consumers to know their perception of freshness and “best before” dates in the case of olive oil. The question “When you buy olive oil, do you check the best before date?” was answered by 439 consumers. More than 65% of consumers always check the best-before date before purchasing. From these respondents, 17% of them check that the best-before date has not passed while the other 48% buy those bottles labeled with the most distant “best before” date for a better guarantee of purchasing a fresh product. A significant percentage of consumers (35%), however, do not check the “best before” date prior to the purchase, though 22% of them think that this term is a good indicator of a fresh product. In summary, around 70% of consumers provided answers that denoted a perception of the “best before” date marking as a parameter associated with the freshness of the oil.

### 3.3. Sensory Quality and Quality Management

The results of the question described above pointed out that the “best before” date was relevant for consumers in general terms, although the concept of this date may be unclear for many of them. On the other hand, the relationship of date marking and the sensory quality was selected as one the most relevant topics in virgin olive oil marketing in the previous interviews with olive oil actors. For that reason, the survey also analyzed the opinion that olive oil actors (producers, analysts, exporters/importers, regulatory bodies) have about the relationship between “best before” date and sensory quality. Thus, the question was “Does the ‘best before’ date guarantee the quality of virgin olive oil when reaching the consumer?” and it was answered by 146 actors as they were supposed to have better knowledge on this matter. The answers show that 63% of the actors do not think “best before” dates guarantee virgin olive oil quality, though 37% of them agree with a relationship between “best before” date and the sensory quality of the purchased virgin olive oil. These answers may denote that they think that the “best before” date is an important variable, although factors may be considered in the transport and distribution of the product for a better guarantee. It was a conflicting matter for respondents since 24% of them added a free text to explain their viewpoints. Thus, some opinions mentioned that “There are factors that affect the shelf-life such as storage conditions, antioxidant contents, etc.” and “The best before is too long in many cases”. These comments are in line with the works published by Wang et al. [53], Aparicio-Ruiz et al. [54] and Lobo-Prieto et al. [56]. These publications discussed the difficulty of establishing a proper “best before” date for a guarantee of quality, particularly when more than one year is considered. On the other hand, the lack of consensus in the measurements for establishing a reliable “best before” date may also be related with this perception of a quality guarantee.

The next question of the survey was focused on knowing the best technical aspect to determine the “best before” date. The question “Should the ‘best before’ date be based on the date of bottling or the date of harvesting?” was posed to producers, importers, exporters and regulatory bodies and was answered by 146 people. The majority of the respondents (51%) are of the opinion that the “best before” date should be based on the date of harvesting and 33% on the date of bottling. However, 16% of actors thinks that the best before date should be based on other technical aspects, though they do not describe an alternative but a generic approach. Some comments were provided, such as “different oils and varieties have significantly different profiles and shelf life, and the best by date should be based on some kind of objective measurements taken at the time of bottling”. Other respondents thought that “at least the olive campaign in which olives were harvested and milled should always be on the label” and “Best before should be calculated with reference to the time the oil was analyzed”. The idea of determining the “best before” date as a function of the specific values of objective chemical parameters of the sample instead of a generic fixed time of 24 months from the time of harvesting is in line with the use of the value of pyropheophytins for determining the “best before” date management [54].

The answers on the “best before” date announced the importance that sensory quality has for all the olive oil actors. Thus, virgin olive oil is a product with particular complex production and distribution schemes where the evaluation of intrinsic quality of the product is necessary [56]. The sensory quality of virgin olive oils, focused on their aroma and taste, is determined by a regulated sensory assessment, so called Panel Test [7,20,57], that classifies virgin olive oils into categories or grades (extra virgin, virgin, ordinary and lampante) and is accepted in national and international norms worldwide [3,4]. In fact, IOC trade standards [3], the objective of which is achieving uniformity in international trade, describes the limits of the parameters connected to physical-chemical and organoleptic characteristics of olive oil. The survey aimed to know the opinion of the actors that are more directly involved in the market with respect to the trade standards protecting quality. They are producers, analysts, retailers, importers, exporters and professionals working at regulatory bodies. The question of the questionnaire was: “Are the current regulation and standard methods efficient in protecting consumers and favouring fair competition?”. From the respondent, most of them (77%) answered that the current regulation and methods are efficient (16%) or partially efficient (61%), while 26% of respondents pointed out the need of significant improvement. A high percentage (43%) of respondents of the latter group expressed their viewpoints with comments. Thus, some of the respondents referred to the evolution of parameters over time: “We need to separate quality standards from authenticity standards, and acknowledge what changes over time and what does not”. Thus, some of the comments were related with the interpretation of the quality parameters in the particular case of a quality decay because of an extended storage, which is a different situation from a non-efficient quality control of the product before bottling [54].

The survey also puts the focus on sensory quality and their control by means of the organoleptic assessment. Thus, the survey addressed a study of the opinions on the Panel Test, in which first question was: “Are sensory assessment (panel tests) objective enough to protect virgin olive oil quality?”. This question was answered by 123 actors (producers, retailers, analysts, importer, exporter and persons working at regulatory bodies). An ample set of them (74%) answered affirmatively. From the respondents, 42% mentioned some drawbacks that result in erroneous classifications, for instance, qualifying virgin olive oils as extra virgin olive oils and vice versa. The reported information was also analyzed according to whether the actors were from or outside Europe. Non-European actors—mainly from importer countries—are more sensitive to possible drawbacks of the Panel Test. A full positive answer (“sensory assessment is enough to protect virgin olive oil quality”) was given by 34% European vs. 26% non-European actors.

The same actors (123) answered the following question on the same matter: “Would you remove the Panel Test from the current regulations/trade standards?”. The vast majority of respondents (90%) would not remove Panel Test from regulations—“I think Panel test is the best” says a respondent—although 54% show some remarks on its application and 43% of the actors opine that Panel Test should be revised for a better implementation. Comments, in general, emphasize that the standard analytical methods cannot substitute the sensory assessment with success today. They argued that “There are far more cases in which a virgin olive oil with defect has an acceptable chemical profile” or “Current chemical standards do not appear to have a consistent and objective relationship with sensory findings”.

Finally, the survey asked the same actors another alternative for the Panel Test with the following question: “Concerning sensory quality of virgin olive oil categories, would you apply an alternative method that is not based on the Panel Test?”. This question was answered by 120 actors. Only 43% of the respondents were completely sure to look for an alternative method while 34% think that Panel Test has no alternative. A substantial number of actors (23%) have an intermediate opinion as they think that the hypothetical alternative should be applied only as a screening method prior to a sensory assessment by Panel Test. The actors illustrated this last viewpoint with some interesting comments: “Experience and several studies have shown inconsistencies among panels, even the same panel with the same oil”, “It is known that the virgin olive oil sensory properties change over time—how are we to enforce what is bottled versus what is on the shelf within one year?”, “The adoption of rapid, objective and reproducible analytical methods should be the goal of future work”. Thus, the responses seem to indicate that there is agreement that the new strategies could be applied as a supportive analytical tool rather than a substitute for the panel test. The survey was also interested in knowing the viewpoint of analysts and staff working at regulatory bodies about the use of techniques other than chromatography in the laboratories for the control of olive oil purity and quality. The question “Would you substitute/supplement chromatographs for/with other instrumentations (e.g., NMR, FTIR) in your labs for quality/purity control?” was answered by 109 actors (analysts and regulatory bodies). More than one half of the respondents (54%) answered affirmatively, while 38% of actors think that further research is needed to evaluate their analytical quality parameters (e.g., detection limit, repeatability, robustness), and only 8% of them have a negative viewpoint of the application of these techniques, mainly due to the high cost of the instruments. The experience of the respondents in the use of NIR spectrometers for some basic parameters (e.g., free acidity) may have influenced the answers. On the other hand, it can be argued that these answers may evolve over time depending on the distribution of new spectroscopic strategies in the future.

## 4. Conclusions

The questions were selected for posing aspects whose answers were not evident or unanimous. The fact that the questions kindle some debate were proved by the percentages computed for the possible answers. Thus, a question with percentages of 95% for one of the possible answers could mean that there is consensus and probably no debate on that topic. However, the selected answers provided percentages of 70% or less, which means that not all the respondents have the same opinion. Some relevant conclusions can be extracted from the olive oil survey. Thus, most of consumers (67%) agree with the importance of PDO and this percentage was lower (56%) for the other olive oil actors. The higher traceability standards carried out by the PDO seem to be appreciated by consumers. In fact, there is a certain willingness to pay more for a perfected traceability system and this willingness was expressed by 74% of consumers. On the other hand, the geographical traceability was regarded as a relevant objective for traceability followed by a better control of adulteration. Regarding the possibility of a centralized system of traceability, 80% of actors would agree with this option, although there is some reluctance for sharing data or paying for a databank. The rights to have access to an open data space and the possible use of these data seem to raise issues in the productive sector.

Regarding quality, only the half of respondents agree with a category beyond extra virgin olive oil (premium oil). It seems that the consumers’ perception of a “best before” date marked on the label is positive and it is associated with freshness. However, it is questioned to what extent this date is a guarantee of freshness. Thus, many respondents agree that they care about the “best before” date when purchasing olive oil but they perceive that it is not a total guarantee of quality. In fact, this opinion agrees with the fact that many other factors may alter quality. This shows that the determination of “best before” date is still an ongoing hot topic.

Regarding the standard method for the organoleptic assessment, most of respondents (90%) do not agree with removing panel tests as a mandatory analysis, although many of them agree with some improvements (43%) and with the implementation of alternative methods (80%). Some (33%) of respondents regard this alternative method as screening. These answers show a general acceptance of the need for developing methods for supporting panel test. Regarding the development of other methods for olive oil authenticity control in addition to those described in the current norms, half of respondents would try non-targeted methods (e.g., spectroscopic methods) as an alternative control, although at the same time they seem to demand some improvements. There is also some general agreement in the interest of improving the current methods.

The answers to these questions provide a basis for general information on which points there is a dispute or a basic agreement. This information base can in turn form the basis for an incentive to develop solutions for the main challenges, such as the development and implementation of supportive methods for quality assessment and better handling of the “best before” date. Likewise, this information can be used to address future survey studies to trace the evolution and achievement of these challenges.

## Data Availability

No new data were created or analyzed in this study. Data sharing is not applicable to this article.

## Supplementary Materials

The following supporting information can be downloaded at: https://www.mdpi.com/article/10.3390/foods11071045/s1, Questions of survey form launched to olive actors.

## References

[1] Fraga H., Moriondo M., Leolini L., Santos J.A. (2021). Mediterranean olive orchards under climate change: A review of future impacts and adaptation strategies. Agronomy.

[2] IOC International Olive Council (2021). World Olive Oil and Table Olive Figures. Production. Madrid Spain. https://www.internationaloliveoil.org/what-we-do/economic-affairs-promotion-unit/#figures.

[3] IOC International Olive Council (2021). Trade Standard Applying to Olive Oils and Olive Pomace Oils. COI/T.15/NC No 3/Rev. 17. Madrid, Spain. https://www.internationaloliveoil.org/wp-content/uploads/2021/11/COI-T15-NC3-REV-17_ENK.pdf.

[4] EEC (1991). Commission Regulation (EEC) No 2568/91 of 11 July 1991 on the characteristics of olive oil and olive-residue oil and on the relevant methods of analysis. Consolidated Text. 01991R2568—EN—20.10.2019—032.001. Off. J. Eur. Community.

[5] Tena N., Wang S.C., Aparicio-Ruiz R., García-González D.L., Aparicio R. (2015). In-depth assessment of analytical methods for olive oil purity, safety, and quality characterization. J. Agric. Food Chem..

[6] García-González D.L., Tena N., Romero I., Aparicio-Ruiz R., Morales M.T., Aparicio R. (2017). A study of the differences between trade standards inside and outside Europe. Grasas Aceites.

[7] García-Gonzalez D.L., Aparicio R., Aparicio-Ruiz R., Morin J.F., Lees M. (2018). Olive oil. Food Integrity Handbook: A Guide to Food Authenticity Issues and Analytical Solutions.

[8] Romero N., Saavedra J., Tapia F., Sepúlveda B., Aparicio R. (2016). Influence of agroclimatic parameters on phenolic and volatile compounds of Chilean virgin olive oils and characterization based on geographical origin, cultivar and ripening stage. J. Sci. Food Agric..

[9] Aparicio-Ruiz R., Morales M.T., Aparicio R. (2019). Does authenticity of virgin olive oil sensory quality require input from Chemistry?. Eur. J. Lipid Sci. Technol..

[10] Mili S. (2016). Value chain dynamics of agri-food exports from southern Mediterranean to the European Union: End-market perspective. Int. J. Food Syst. Dyn..

[11] Mansouri F., Ben moumen A., Belhaj K., Richard G., Fauconnier M.L., Sindic M., Caid H.S., Elamrani A. (2018). Effect of crop season on the quality and composition of extra virgin olive oils from Greek and Spanish varieties grown in the Oriental region of Morocco. Emirates J. Food Agric..

[12] Di Lecce G., Piochi M., Pacetti D., Frega N.G., Bartolucci E., Scortichini S., Fiorini D. (2020). Eleven monovarietal extra virgin olive oils from olives grown and processed under the same conditions: Effect of the cultivar on the chemical composition and sensory traits. Foods.

[13] Giuffrè A.M. (2013). Influence of cultivar and harvest year on triglyceride composition of olive oils produced in Calabria (Southern Italy). Eur. J. Lipid Sci. Technol..

[14] Ollivier D., Artaud J., Pinatel C., Durbec J.P., Guérère M. (2003). Triacylglycerol and fatty acid compositions of French virgin olive oils. Characterization by chemometrics. J. Agric. Food Chem..

[15] Giuffrè A.M. (2014). The effects of cultivar and harvest year on the fatty alcohol composition of olive oils from Southwest Calabria (Italy). Grasas Aceites.

[16] Skiada V., Agriopoulou S., Tsarouhas P., Katsaris P., Stamatelopoulou E., Varzakas T. (2020). Evaluation and origin discrimination of two monocultivar extra virgin olive oils, cultivated in the coastline part of north-western Greece. Appl. Sci..

[17] Giuffrè A.M. (2013). Influence of harvest year and cultivar on wax composition of olive oils. Eur. J. Lipid Sci. Technol..

[18] Jimenez-Lopez C., Carpena M., Lourenço-Lopes C., Gallardo-Gómez M., Lorenzo J.M., Barba F.J., Prieto M.A., Simal-Gandara J. (2020). Bioactive compounds and quality of extra virgin olive oil. Foods.

[19] Genovese A., Caporaso N., Sacchi R. (2021). Flavor chemistry of virgin olive oil: An overview. Appl. Sci..

[20] IOC International Olive Council (2018). Sensory Analysis of Olive Oil–Method for the Organoleptic Assessment of Virgin Olive Oil. COI/T.20/Doc. No 15/Rev. 10. Madrid, Spain. https://www.internationaloliveoil.org/wp-content/uploads/2019/11/COI-T20-Doc.-15-REV-10-2018-Eng.pdf.

[21] Martín-Peláez S., Covas M.I., Fitó M., Kušar A., Pravst I. (2013). Health effects of olive oil polyphenols: Recent advances and possibilities for the use of health claims. Mol. Nutr. Food Res..

[22] Vargas A.J., Neuhouser M.L., George S.M., Thomson C.A., Ho G.Y., Rohan T.E., Kato I., Nassir R., Hou L., Manson J.E. (2016). Diet quality and colorectal cancer risk in the Women’s Health Initiative Observational Study. Am. J. Epidemiol..

[23] Cicerale S., Lucas L., Keast R. (2012). Antimicrobial, antioxidant and anti-inflammatory phenolic activities in extra virgin olive oil. Curr. Opin. Biotechnol..

[24] Schwingshackl L., Morze J., Hoffmann G. (2020). Mediterranean diet and health status: Active ingredients and pharmacological mechanisms. Br. J. Pharmacol..

[25] Visioli F., Davalos A., López de las Hazas M.C., Crespo M.C., Tomé-Carneiro J. (2020). An overview of the pharmacology of olive oil and its active ingredients. Br. J. Pharmacol..

[26] Virruso C., Accardi G., Colonna-Romano G., Candore G., Vasto S., Caruso C. (2014). Nutraceutical properties of extra-virgin olive oil: A natural remedy for age-related disease?. Rejuvenation Res..

[27] Fotopoulos C., Krystallis A. (2001). Are quality labels a real marketing advantage? A conjoint application on Greek PDO protected Olive Oil. J. Int. Food Agribus..

[28] Cafarelli B., Sala P.L., Pellegrini G., Fiore M. (2017). Consumers’ preferences investigation for extra virgin olive oil basing on conjoint analysis. Rivista di Studi sulla Sostenibilita.

[29] Aparicio-Ruiz R., García-González D.L., Lobo-Prieto A., Aparicio R. (2019). Andalusian protected designations of origin of virgin olive oil: The role of chemical composition in their authentication. Eur. J. Lipid Sci. Technol..

[30] Bendini A., Cerretani L., Carrasco-Pancorbo A., Gómez-Caravaca A.M., Segura-Carretero A., Fernández-Gutiérrez A., Lercker G. (2007). Phenolic molecules in virgin olive oils: A survey of their sensory properties, health Effects, antioxidant activity and analytical methods. An overview of the last decade. Molecules.

[31] Duman S., Guldas M. (2008). An impact assessment of origin labeling on table olive and olive oil demand. Acta Hortic..

[32] Erraach Y., Sayadi S., Gómez A.C., Parra-López C. (2014). Consumer-stated preferences towards Protected Designation of Origin (PDO) labels in a traditional olive-oil-producing country: The case of Spain. New Medit.

[33] Menapace L., Colson G., Grebitus C., Facendola M. (2011). Consumers’ preferences for geographical origin labels: Evidence from the Canadian olive oil market. Eur. Rev. Agric. Econ..

[34] Perito M.A., Sacchetti G., Di Mattia C.D., Chiodo E., Pittia P., Saguy I., Cohen E. (2019). Buy local! Familiarity and preferences for extra virgin olive oil of Italian consumers. J. Food Prod. Mark..

[35] Santosa M., Abdi H., Guinard J.-X. (2010). A modified sorting task to investigate consumer perceptions of extra-virgin olive oils. Food Qual. Prefer. J..

[36] Panico T., Del Giudice T., Caracciolo F. (2014). Quality dimensions and consumer preferences: A choice experiment in the Italian extra-virgin olive oil market. Agric. Econ. Rev..

[37] Aprile M.C., Caputo V., Nayga R.M. (2012). Consumers’ valuation of food quality labels: The case of the European geographic indication and organic farming labels. Int. J. Consum. Stud..

[38] Del Giudice T., Cavallo C., Caracciolo F., Cicia G. (2015). What attributes of extra virgin olive oil are really important for consumers: A meta-analysis of consumers’ stated preferences. Agric. Food Econ..

[39] Dekhili S., Sirieeix L., Cohen R. (2011). How consumers choose olive oil: The importance of origin cues. Food. Qual. Prefer..

[40] Scarpa R., Del Giudice T. (2004). Market segmentation via mixed logit: Extra-virgin olive oil in urban Italy. J. Agric. Food Ind. Organ..

[41] Dichter E. (1962). The world customer. Harv. Bus. Rev..

[42] Bernabéu R., Díaz M. (2016). Preference for olive oil consumption in the Spanish local market. Span. J. Agric. Res..

[43] Carlucci D., De Gennaro B., Roselli L., Seccia A. (2014). E-commerce retail of extra virgin olive oil: An hedonic analysis of Italian SMEs supply. Br. Food J..

[44] Inanc Güney O., Sangün L. (2017). Olive oil consumption attitudes as a healthy food: A survey study on young consumers. Agro Food Ind. Hi-Tech.

[45] Romo-Muñoz R.A., Cabas-Monje J.H., Garrido-Henrríquez H.M., Gil J.M. (2017). Heterogeneity and nonlinearity in consumers’ preferences: An application to the olive oil shopping behavior in Chile. PLoS ONE.

[46] Salazar-Ordóñez M., Rodríguez-Entrena M., Cabrera E.R., Henseler J. (2018). Survey data on consumer behaviour in olive oil markets: The role of product knowledge and brand credence. Data Br..

[47] EC European Commission (2021). Consolidated Version (26.05.2021) of Regulation (EC) No 178/2002 of the European Parliament and of the Council of 28 January 2002 Laying Down the General Principles and Requirements of Food Law, Establishing the European Food Safety Authority and Laying Down Procedures in Matters of Food Safety. https://eur-lex.europa.eu/legal-content/ES/TXT/?uri=CELEX%3A02002R0178-20210526&qid=1646147440063.

[48] EC European Commission (2010). Guidance on the Implementation of Articles 11, 12, 14, 17, 18, 19 and 20 of Regulation (EC) No 178/2002 on General Food Law Conclusions of the Standing Committee on the Food Chain and Animal Health. 20 January 2010 Brussels. https://ec.europa.eu/food/system/files/2016-10/gfl_req_guidance_rev_8_en.pdf.

[49] Tena N., Aparicio-Ruiz R., Koidis A., García-González D.L., Didier M., Ramesh R.C. Analytical tools in authenticity and traceability of olive oil. Food Traceability and Authenticity: Analytical Techniques.

[50] European Commission (EC) (2020). Communication from the Commission to the European Parliament, the Council, the European Economic and Social Committee and the Committee of the Regions. A European Strategy for Data. Brussels, 19.2.2020 COM 66 final. https://ec.europa.eu/info/sites/default/files/communication-european-strategy-data-19feb2020_en.pdf.

[51] Ballco P., Gracia A. (2020). Do market prices correspond with consumer demands? Combining market valuation and consumer utility for extra virgin olive oil quality attributes in a traditional producing country. J. Retail. Consum. Serv..

[52] Marakis G., Gaitis F., Mila S., Papadimitriou D., Tsigarida E., Mousia Z., Karpouza A., Magriplis E., Zampelas A. (2021). Attitudes towards Olive Oil Usage, Domestic Storage, and Knowledge of Quality: A Consumers’ Survey in Greece. Nutrients.

[53] Wang S., Moscatello B., Flynn D. (2013). Consumer attitudes on olive oil. Publication and Reports of UC Davis Olive Center.

[54] Aparicio-Ruiz R., Aparicio R., García-González D.L. (2014). Does “best before” date embody extra-virgin olive oil freshness?. J. Agric. Food Chem..

[55] Lobo-Prieto A., Tena N., Aparicio-Ruiz R., García-González D.L., Sikorska E. (2020). Monitoring virgin olive oil shelf-life by fluorescence spectroscopy and sensory characteristics: A multidimensional study carried out under simulated market conditions. Foods.

[56] Lobo-Prieto A., Tena N., Aparicio-Ruiz R., Morales M.T., García-González D.L. (2020). Tracking sensory characteristics of virgin olive oils during storage: Interpretation of their changes from a multiparametric perspective. Molecules.

[57] Angerosa F., Campestre C., Aparicio R., Harwood J. (2013). Sensory quality: Methodologies and application. Handbook of Olive Oil: Analysis and Properties.

