# Supplementary material for: An International Survey on Olive Oils Quality and Traceability: Opinions from the Involved Actors
Source: Foods. 2022 Apr 5;11(7):1045. doi: 10.3390/foods11071045 (PMC8997808; doi:10.3390/foods11071045)
Supplement: Supplementary file 1 [file foods-11-01045-s001.zip › foods-1644566-supplementary.pdf]

SUPPLEMENTARY INFORMATION

An International Survey on Olive Oils Quality and Traceability: Opinions from the Involved Actors

Ramón Aparicio-Ruiz, Noelia Tena Pajuelo, Diego L. García-González

SURVEY FORM

GENERAL INFORMATION

**1.- Please, indicate your country (*Free text*):**
**2.- Please, indicate your professional sector (*Multi-answer Multiple Choice*):**<br>• **Farmer**<br>• **Producer**<br>• **Retailer**<br>• **Importer**<br>• **Exporter**<br>• **Analyst**<br>• **Regulatory body**<br>• **Consumer**<br>• **Others (please, specify) (*Free Text*)**

PRODUCTION

1.- Would you be willing to adopt a high-density production system (~200 trees/ha or 80 trees/ac) that would be mechanically harvested and pruned? (*Single-answer Multiple Choice*)

- **No, I wouldn't.**
- **Yes, I would.**
- **I would need to study this option further.**

Please, provide any comment about your opinion in this regard:

Question for farmers

2.- In phytosanitary terms, which of the following tasks should be further investigated in the future? Please select one:

- **Protection against the bacterium Xylella fastidiosa.**
- **Protection against Verticillium infection.**
- **Integrated control of olive tree diseases.**
- **Other phytosanitary problems:**

Question for farmers

3.- From the point of view of irrigation management in olive orchards, which of the following tasks should be further investigated in the future? Please select one:

- **New olive tree varieties very resistant to drought.**
- **New varieties of olive trees for high density orchards.**
- **Irrigation of orchards with saline water.**
- **Irrigation management and planning in potential irrigation areas.**
- **Soil management in runoff and erosion of olive trees areas.**

- **Others (please, specify):**

Question for farmers

4.- Do you think that your oil mill would improve, if the research is focused on:

- **Improving the quality during the extraction**.
- **Improving mixers to be cleaner, more efficient and rapid.**
- **The control of fermentation in new vertical decanters/tanks.**

Please, would you like to contribute with a free text?

Question for farmers

5.- In your opinion, how should be paid the olives to farmers?

- **By weight.**
- **By weight associated with the oil yield of olives.**
- **By weight associated with the oil yield of the olive and the free acidity.**
- **By weight associated with the oil yield of the olive, the free acidity and the sensory quality.**
- **By others (please, specify):**

Question for farmers
**6.- A Protected Designation of Origin (PDO) system differentiate a product from other similar products and therefore it means a protection for certain foodstuffs with a recognised know-how. Do you agree with this statement? (*Single-answer Multiple Choice*)**<br><br>• **Very much**<br>• **A little**<br>• **No at all, PDO is actually a kind of trademark**<br>• **Do not know.**<br><br>***Please, would you like to contribute with a free text? (Optional) (Free Text)***<br><br>*Question for producers, retailers, importers, exporters, analysts, regulatory bodies and consumers.*
**7.- In the particular case of virgin olive oil, do you understand what a PDO means? (*Single-answer Multiple Choice*)**<br><br>• **Yes**<br>• **No**<br>• **Not sure**<br><br>***Please, would you like to contribute with a free text? (Optional) (Free Text)***<br><br>*Question for consumers.*
**8.- In case of a Yes answer in Question 2: Please could you explain what do you understand?**<br><br>*Question for consumers.*

TRADE AND REGULATION

9.- Are the current regulation and standard methods efficient in protecting consumers and favouring fair competition?

- **Yes, they are efficient in this task.**
- **Partly, but some improvements could be made.**
- **No, they need significant improvements (e.g. limits of some fatty acids and sterols).**

Any comment (optional) (*Free Text*):

Question for producers, retailers, importers, exporters, analysts, regulatory bodies.

10.- In a global viewpoint, would you agree to the establishment of a single global norm at world level (including regional governments) to ease and promote commercialization and transactions?

- Yes, I would.
- No, I would not.

Please, explain why:

Questions for retailers, exporters, importers, analysts and regulatory bodies.

TRACEABILITY*

* Regulation EC/178/2002 defines traceability as the ability to trace and follow food, feed, and ingredients through all stages of production, processing and distribution. Ability to trace the history, application or location of an entity through the food chain from its **geographical origin** to the point where it reaches the consumer, and *vice versa*.
**11.- In your opinion, what are the three main objectives of the implementation of an efficient traceability system for olive oil and virgin olive oil? (Multiple-answer multiple choice up to three).**<br><br>• **Detection of pesticides and other contaminants**<br>• **Geographical Identification (distinction of different production areas)**<br>• **Detection of critical points affecting quality**<br>• **Reproducibility in the production (between crops, extraction systems)**<br>• **Detection of critical points affecting safety**<br>• **Detection of adulteration of olive oil**<br>• **Protection of Protected Designation of Origin (PDO)**<br>• **Additional guarantee of the product to gain consumer confidence (competitive product differentiation)**<br>• **Others (Free text)**<br>•<br>**If you wish, could you explain briefly the importance of the main objectives that you have selected in the previous question? (optional): (*Free Text*)**<br><br>*Question for producers, retailers, importers, exporters, analysts, regulatory bodies.*
**12.- At which of the following levels should a traceability system be implemented? (Single-answer Multiple Choice)**<br><br>• **At regulatory level**<br>• **At production level**<br>• **At supply-chain or commercial level**<br>• **All above.**<br><br>***Please, would you like to contribute with a free text?***<br><br>*Question for retailers, exporters, importers, analysts and regulatory bodies.*
**13.- As a consumer, are you willing to pay more for a virgin olive oil with additional assurance regarding traceability? (Single-answer Multiple Choice)**<br><br>• **Yes, because I feel more confident with the product.**<br>• **No, it is quite expensive already.**<br><br>***Please, would you like to contribute with a free text? (Optional) (Free Text)***<br><br>*Question for consumers***.**

14.- With respect to traceability and your opinion, what are the problems of implementing a traceability system in olive oil production? (please indicate three challenges)

- **Lack of understanding of the use of implementing traceability.**
- **Lack of analytical tools.**
- **Lack of guidance on how to implement the traceability.**
- **Costs of implementing the traceability outweigh the benefits.**
- **Complexity of the product (number of categories, many actors in distribution).**
- **If there are problems other than the ones mentioned in the above question, please state them: (Free Text)**

Question for retailers, importers, exporters, regulatory bodies.

15.- In the case of Geographical Origin traceability, what is the main function a traceability system should perform? (Single-answer Multiple Choice)

- **Fast detection of adulterated oils (safety crisis in a particular location)**
- **Protect traditional production (e.g. Protected Designation of Origin, PDO)**
- **To distinguish different production areas (e.g. European and non-European oils)**

Please, specify which area(s) if you wish (optional): (Free text).

Question for producers, analysts, regulatory bodies.

16.- Given that there is an European regulation and other norms that force traceability systems on food companies, do you think that the traceability system should be centralized in a national/International body? (Single-answer Multiple Choice)

- **Yes, that would provide a better harmonisation of traceability systems.**
- **Yes, but the centralised traceability system should be IN ADDITION to the one implemented by the company as the objectives are different.**
- **No, each company/producer should implement domestic measures to ensure compliance with traceability requirement adapted to their capabilities.**

Please, would you like to contribute with a free text? (Optional) (Free Text)

Question for producers, analysts, exporters, importers, regulatory bodies.

17- With respect to traceability and your opinion, would you be willing to provide your recorded data to a network of producers for a better synergy in traceability? (Single-answer Multiple Choice)

- **Yes, it would improve the traceability system and consistency and transparency of data exchange would be positive.**
- **No, that is against our interests.**
- **It depends on the conditions of data sharing.**

If you answer that it depends on the conditions of data sharing, please specify under which conditions it could be acceptable to you (Optional): (Free Text)

Question for producers, importers, exporters, analysts, regulatory bodies.

QUALITY AND CONSUMERS

According to the International Olive Council the definitions of olive oil and virgin olive oil are:

Olive oil is the oil consisting of a blend of refined olive oil and virgin olive oils fit for consumption as they are. It has a free acidity, expressed as oleic acid, of not more than 1 gram per 100 grams and its other characteristics correspond to those fixed for this category in this standard.

Virgin olive oil is the oil obtained from the fruit of the olive tree solely by mechanical or other physical means under conditions, particularly thermal conditions, that do not lead to alterations in the oil, and which have not undergone any treatment other than washing, decantation, centrifugation and filtration.

Extra virgin olive oil has a free acidity, expressed as oleic acid, of not more than 0.8 grams per 100 grams, and the other characteristics of which correspond to those fixed for this category in this standard.

International Olive Council (IOC) Document COI/T.15/NC No 3 Trade standard applying to olive oils and olive-pomace oils, Madrid.

18.- When you buy olive oil, do you understand the label with respect to the different quality categories?

- **Yes, I do.**
- **No, I do not.**
- **Not totally, I have some doubts.**

In case you do not completely understand the label, could you please suggest some improvements that could be made to make the information clearer? (optional): (*Free Text*)

Question for consumers

19.- According to your experience, do you think that the current methods for the virgin olive oil quality assessment are useful?

- **Yes, they are.**
- **Yes, but they have some drawbacks that result in erroneous classification.**
- **No, they are not.**

If negative, please give reasons:

Question for producers

20.- Do you think that a new quality category higher than extra virgin olive oil is necessary or it might bring more confusion? (Single-answer Multiple Choice)

- **Yes, it is necessary to distinguish those oils that are "premium" from those that are not that good.**
- **No, it would be confusing and it is not needed.**

Any further comment (optional) (*Free Text*):

Question for consumers.

21.- In case of an affirmative answer, should this new category be included in labelling regulations or should it be optional instead? (Single-answer Multiple Choice)

- **It should be included in regulations.**
- **No, it should be a commercial initiative and optional.**
- **I am not sure.**

Please, would you like to contribute with a free text? (Optional) (Free Text)

Question for consumers.

22.- When you buy olive oil, do you check the "best before" date?

- **Yes, I always check that the "best before" date is as distant as possible from the current date to have a fresh product**
- **Yes, always, but I only check that the "best before" date is not surpassed**
- **No, I never thought of checking this before, but I think it is a good indicator of freshness**
- **No, I don't think the "best before" date guarantees the freshness of the olive oil.**

Please, would you like to contribute with a free text? (Optional) (Free Text)

Question for consumers.

Sensory Assessment (Panel test): Standard method to determine sensory quality of virgin olive oil consisting in the examination of the organoleptic attributes of a product by the sense organs. The sensory assessment is carried out by a group of assessors who have been specially selected and trained (EU, No 2568/91).
**23.- Are sensory assessments (panel tests) objective enough to protect virgin olive oil quality? (*Single-answer Multiple Choice)*** <br>• **Yes, it is.**   • **Yes, but they have some drawbacks that result in erroneous classification.**   • **No, it is not.** <br>**If negative, please give reasons (optional) (*Free Text*):** <br>*Question for producers, retailers, importers, exporters, analysts, regulatory bodies.*
**24.- Concerning sensory quality of virgin olive oil categories, would you apply an alternative method that is not based on the Panel Test? (Single-answer Multiple Choice)** <br>• **Yes, I would.**   • **No, I would not.**   • **Yes, but only as a screening method prior to sensory assessment by panel test.** <br>**Please, provide any further comment to explain the answer (*Free Text*):** <br>*Question for producers, retailers, importers, exporters, analysts, regulatory bodies.*
**25.- Would you remove the Panel Test from the current regulations/trade standards? (Single-answer Multiple Choice)** <br>• **Yes, it provides more problems than solutions.**   • **No, although I would revise its implementation to avoid the criticisms.**   • **No, removing it would mean a poorer quality assurance.** <br>*Question for producers, retailers, importers, exporters, analysts, regulatory bodies.*
**26.- Does the "best before" date guarantee the quality of virgin olive oil when reaching the consumer?** <br>• **Yes, it does.**   • **No, it does not.** <br>**Any further comment (optional): (*Free Text*)** <br>*Question for producers, importers, exporters, analysts, regulatory bodies.*
**27.- Should the "best before" date be based on the date of bottling or the date of harvesting?** <br>• **Date of bottling**   • **The date of harvesting**   • **Any other criterion (*Free Text*)** <br>*Question for producers, importers, exporters, regulatory bodies.*

AUTHENTICATION

28.- Would you substitute/supplement chromatographs for/with other instrumentations (e.g. NMR, FTIR) in your labs for quality/purity control? (Single-answer Multiple Choice)

- **Yes, they can facilitate lab work.**
- **No, further research is needed.**
- **No, I think that they cannot be an alternative.**

Please, would you like to contribute with a free text? (Optional) (Free Text)

Question for analysts, regulatory bodies.

29.- Would you be interested in having access to an analytical database dedicated to olive oil? (Single-answer Multiple Choice)

- **Yes**
- **No**

Please, would you like to contribute with a free text? (Optional) (Free Text)

Question for producers, analysts, regulatory bodies.

30.- If the answer was affirmative: Would you pay for access to this database?

- **Yes**
- **No**

Please, would you like to contribute with a free text? (Optional) (Free Text)

Question for producers, analysts, regulatory bodies.

31.- What are in your opinion the main difficulties/challenges in the field of olive oil analysis?

Question for analysts, regulatory bodies

FOOD INTEGRITY

This project has received funding from the European Union's Seventh Framework Programme for research, technological development and demonstration under grant agreement no 613688
